# Potential effectiveness of war-adapted brief prolonged exposure for PTSD prevention: preliminary study among Nova Festival survivors during ongoing conflict

**DOI:** 10.1192/bjo.2026.12116

**Published:** 2026-10-16

**Authors:** Nitsa Nacasch, Hadas Afgin, Netta Shoenfeld, Ilanit Wul, Gay Meschiany, Edna B. Foa, Raz Gross, Nadav Goldental, Gal Levi, Joseph Zohar, Mark Weiser, Hagit Cohen

**Affiliations:** The Pinchas and Drora Division of Psychiatry, Sheba Medical Center, Tel HaShomer, Israel; Department of Psychiatry, Tel Aviv University School of Medicine, Tel Aviv, Israel; Clinical Psychology, University of Pennsylvania, Philadelphia, USA; Department of Psychology, Ben-Gurion University of the Negev, Beer-Sheva, Israel; Post-Trauma Center, Sheba Medical Center, Tel Aviv University, Israel; Faculty of Health Sciences, Ben-Gurion University of the Negev, Israel & Anxiety and Stress Research Unit, Be’erot Mental Health Center, Beer-Sheva, Israel

**Keywords:** 7 October 2023 attack, PTSD prevention, early intervention, prolonged exposure, substance use

## Abstract

**Background:**

The 7 October 2023, terrorist attack at the Nova Festival in southern Israel caused deaths, injuries, hostage-taking and prolonged life-threatening exposure, creating an urgent need for early interventions to reduce post-traumatic morbidity. Although prolonged exposure is an established post-traumatic stress disorder (PTSD) treatment, its feasibility and benefit during ongoing threat remain unclear.

**Aims:**

This preliminary study evaluated a war-adapted brief prolonged exposure protocol (WBPE) delivered shortly after trauma in Nova Festival survivors with acute stress disorder.

**Method:**

Forty survivors completed the WBPE and were assessed at baseline and immediately post treatment. A subsample of 27 WBPE completers and 18 untreated wait-list controls was reassessed after 5 months. Outcomes were PTSD (PDS-5), depression (PHQ-9) and anxiety (GAD-7) symptom severity.

**Results:**

WBPE was associated with significant reductions in PTSD, depression and anxiety immediately after treatment. At 5 months, WBPE participants showed greater reductions in PTSD and depressive symptom severity and lower PTSD and depression symptom scores than controls, with large effect sizes. Anxiety symptoms improved in both groups, consistent with possible natural recovery. Clinically significant depressive symptoms (48.15 *v*. 77.78%) and anxiety symptoms (37.04 *v*. 72.22%) were less frequent in WBPE participants than controls, whereas probable PTSD rates did not differ significantly. Substance use during the study period was associated with poorer outcomes and smaller symptom reductions.

**Conclusions:**

WBPE appears feasible and potentially beneficial under wartime conditions, with meaningful improvements in PTSD and depression despite persistent threat. Substance use was linked to poorer recovery and should be systematically assessed in post-trauma care.

On 7 October 2023, attendees of the Nova Festival in southern Israel were subjected to an unprecedented terrorist attack during the early morning hours. Thousands of young participants, many of whom were under the influence of recreational drugs, witnessed mass fatalities, severe injuries and the abduction of peers, while some sustained physical injuries themselves. Substance use during the event may have amplified psychological vulnerability and complicated subsequent recovery trajectories.^1^ After the attack, survivors continued to face persistent threats and trauma reminders, including rocket fire, deaths and injuries among peers or community members and a prolonged hostage crisis, conditions that markedly increased the risk for post-traumatic stress disorder (PTSD).

Unlike most psychiatric disorders, PTSD has a clearly identifiable onset linked to an index trauma. This distinctive feature enables prospective prevention strategies and raises a fundamental question: whether early psychological interventions should be delivered universally or targeted specifically to individuals exhibiting acute symptoms. Meta-analyses indicate that trauma-focused cognitive–behavioural therapy (CBT) confers significant benefits primarily for individuals meeting diagnostic criteria for acute stress disorder (ASD) or PTSD, whereas universal interventions in unscreened populations offer no advantage over usual care.^2,3^ Similarly, subthreshold cases derive minimal benefit from clinical intervention, while CBT, including prolonged exposure and cognitive therapy, effectively prevents chronic PTSD when initiated within 1 month post trauma in diagnosed individuals.^4^ These findings underscore the importance of delivering brief, targeted psychological treatments early in the post-trauma period to mitigate long-term psychiatric morbidity. Specific features of the traumatic event, including injury severity, prolonged exposure and loss of loved ones, are known risk factors for chronic PTSD. When combined with ongoing threat during the post-trauma period, these factors underscore the need for timely and targeted intervention.

Brief prolonged exposure (BPE) therapy, typically administered in four to five sessions within weeks of trauma, has demonstrated superiority over supportive counselling for ASD following single-incident events once imminent danger has subsided.^5–9^ However, Nova survivors faced ongoing wartime conditions, presenting a unique context in which to examine the feasibility and potential preventive effects of BPE under continuous threat. In response, Nacasch and colleagues modified the standard prolonged exposure protocol^10^ to address the unique challenges of wartime trauma. They adapted the treatment rationale and in vivo exposure procedures to accommodate ongoing realistic threat, while expanding emotional processing to include loss and survivor guilt in addition to fear, anger, sadness, shame and guilt. The resulting war-adapted brief prolonged exposure (WBPE) protocol consists of five 90 min sessions delivered 2–4 weeks post attack to individuals diagnosed with ASD. WBPE integrates psychoeducation, imaginal exposure, in vivo exposure and emotional processing, all tailored to the context of persistent war-related stressors.

This preliminary study evaluates the feasibility and potential effectiveness of WBPE for mitigating PTSD, depressive and anxiety symptoms among Nova Festival survivors with ASD. Outcomes were examined immediately after intervention and at 5 months post trauma, with the latter compared against an untreated wait-list control group.

## Method

All procedures contributing to this work complied with the ethical standards of the relevant national and institutional committees on human experimentation and with the Declaration of Helsinki, as revised in 2013. All study procedures were approved by the Institutional Review Board (IRB) at Sheba Medical Center (protocol SMC-0787-23). All participants provided written informed consent.

### Traumatic event

On 7 October 2023, at approximately 06.30 h, a siren alerted attendees of the Nova open-air festival in southern Israel to an incoming rocket attack. Concurrently, thousands of Hamas-affiliated terrorists infiltrated southern Israel, conducting coordinated assaults across more than 20 locations. Using vehicles and paragliders, a large group of attackers surrounded the festival grounds and fired indiscriminately at individuals attempting to flee, employing firearms, grenades and rocket-propelled grenades. Many attendees sought refuge in nearby bomb shelters, bushes and orchards; however, limited cover in the open terrain led to a high number of casualties. Others attempting to escape by vehicle encountered traffic congestion and were subsequently targeted. The attack resulted in extensive civilian casualties, including 364 fatalities and at least 40 hostages. Survivors endured prolonged exposure to life-threatening danger, witnessed mass fatalities and in some cases sustained physical injuries. Many remained in hiding for 8–20 h under continuous gunfire and explosions before rescue. After returning home, participants continued to face ongoing war-related threats and trauma reminders, including frequent rocket attacks, searches for missing peers, death notifications regarding friends killed in the attack and injury or death of comrades during the subsequent military conflict.

### Participants

Within 1 month after the attack, 143 survivors sought treatment at the Cognitive Behavioral Therapy (CBT) Clinic at Sheba Medical Center, either via social media outreach or personal referrals.

**Inclusion criteria:** (a) being a Nova Festival survivor aged ≥18 years; (b) having a diagnosis of ASD confirmed by clinical assessment and a Post-Traumatic Diagnostic Scale–Self Report (PDS-5) score ≥18^11,12^; (c) residing at least 60 km from the Gaza border. The distance criterion was applied because individuals living in the Western Negev continued to experience direct and recurrent threat, including repeated rocket barrages and restrictions preventing a return to their homes. These conditions limited the feasibility and safety of standard in vivo exposure assignments, a core component of prolonged exposure therapy.

**Exclusion criteria**: (a) severe physical injury (Abbreviated Injury Scale ≥3); (b) loss of first-degree relatives during the attack (to control for acute bereavement effects); (c) prior psychiatric history, including prior PTSD.

**Assessment procedures:** all participants completed computerised questionnaires, underwent telephone screening by trained clinicians, and provided detailed trauma histories during in-person evaluations. Baseline measures included demographics, substance use history and trauma-related losses.

### Clinical assessments comprised

Clinical outcomes included PTSD symptom severity assessed with the PDS-5^11,12^; anxiety symptom severity assessed with the generalised anxiety disorder-7 (GAD-7)^13^; and depressive symptom severity assessed with the Patient Health Questionnaire-9 (PHQ-9).^14^ Assessments were completed at baseline, immediately after treatment and at the 5-month follow-up. Additional details are provided in the Supplementary Information.

#### Study design and sample

**Fig. 1:**
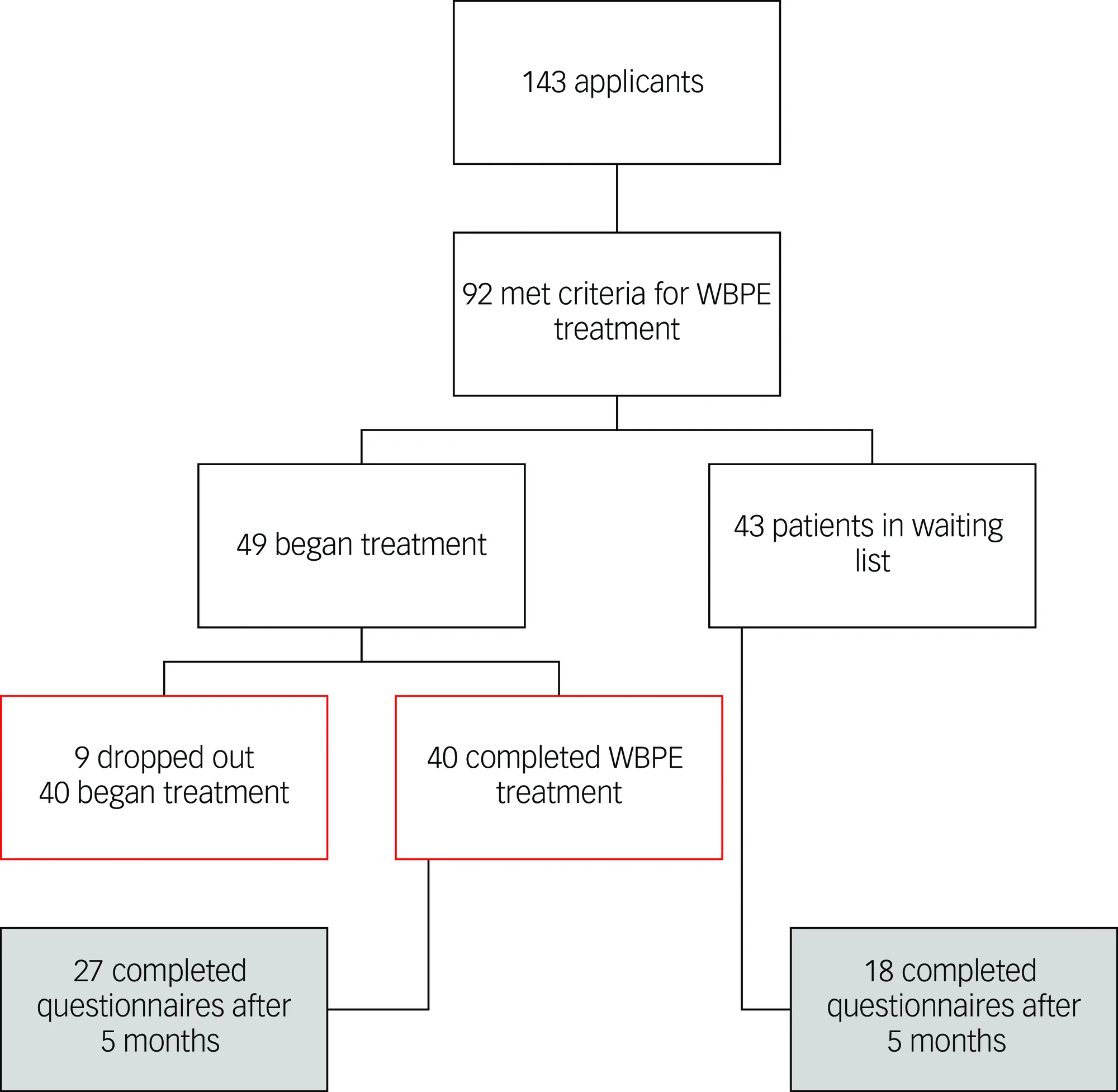
CONSORT flowchart illustrating participant recruitment, eligibility screening and allocation to war-adapted brief prolonged exposure (WBPE) therapy or wait-list control for post-traumatic stress disorder (PTSD) prevention among Nova Festival survivors during ongoing conflict.

Of 143 individuals screened, 92 met eligibility criteria; 49 initiated treatment within 1 month and 43 were assigned to a wait-list comparison group. Of the 49 participants who initiated WBPE, 40 completed the full protocol and 9 discontinued treatment (18.36%). At 5-month follow-up, data were available for 27 treated participants (67.5%) and 18 controls (41.9%) (see Fig. 1 for the participant flow diagram).

#### Wait-list controls

The control group did not receive the WBPE protocol or any study-provided structured mental health intervention during the study period. However, participants could access community-based therapeutic centres established to support Nova Festival survivors. During the study period, two survivors reported receiving eye movement desensitisation and reprocessing (EMDR) therapy, three reported receiving art therapy and three reported a single consultation with a psychologist.

### Intervention

WBPE is a Hebrew-language and wartime-adapted modification of standard prolonged exposure therapy,^10^ tailored to ongoing threat, traumatic grief and survivor guilt. Consistent with emotional processing theory,^15^ which posits that avoidance perpetuates PTSD symptoms, the protocol emphasised gradual exposure to safe trauma-related cues (e.g. listening to trance music, visiting places near shelters). It also targeted maladaptive cognitions following traumatic loss (e.g. ‘I can’t enjoy life when my friends cannot’), encouraged social re-engagement and facilitated emotional processing of guilt, sadness and anger to counteract emotional numbing. The protocol comprised five weekly 90 min sessions.

#### Session 1

The therapist introduces the treatment framework and rationale, emphasising how avoidance maintains PTSD symptoms. A trauma-specific interview is conducted to gather information on demographics, substance use, losses, captivity status and injuries. The index trauma, defined as the most distressing segment of the prolonged (>10 h) event, is identified.

The session also includes psychoeducation regarding common trauma reactions and training in diaphragmatic breathing.

#### Session 2

A personalised in vivo exposure hierarchy is constructed (e.g. darkness, trance music, leaving home near a shelter). Resilience strategies are reinforced (exercise, sleep hygiene, substance reduction, social support and media limitation). The rationale underlying imaginal exposure is presented, followed by 20–30 min of imaginal exposure to the index trauma (audio-recorded). This is followed by 15–20 min of processing, focusing on guilt, sadness, fear, anger and maladaptive cognitions related to ASD and PTSD (e.g. ‘The world is extremely dangerous,’ ‘I am incompetent’). Homework consists of listening to the recording daily and graded in vivo exposures.

#### Sessions 3–5

Each session opens with a 10 min homework review, followed by 20–30 min of imaginal exposure focused on trauma hotspots (introduced from Session 4 onwards) and 15–20 min of emotional processing with increased emphasis on grief and loss integration. In vivo exposure tasks are advanced despite the challenges posed by the persistent war-related threats. Additional details regarding the WBPE protocol are provided in the Supplementary Information.

Therapists included 21 master’s-level clinicians trained in prolonged exposure through a 4-day workshop and weekly supervision; 9 were certified prolonged exposure therapists who had conducted at least 2 prolonged exposure treatments prior to the study.

#### Non-study therapeutic support available to participants

Immediately after the attack, several therapeutic centres were established to provide psychological, rehabilitative and complementary support for Nova Festival survivors and their families. These centres offered nature-based therapeutic environments grounded in a holistic, integrative approach, alongside discussion circles, music circles, complementary therapies, workshops, support groups, social gatherings and memorial events. Participants also had the option of meeting with a psychiatrist. Attendance was voluntary and determined by the survivors themselves. These services were available to participants in both groups and were not part of the study protocol; no individualised long-term treatment plan or structured follow-up was provided within the study framework.

### Statistical analysis

Between-group differences at baseline were examined using a one-way analysis of variance (ANOVA) for continuous variables and Pearson χ^2^ tests for categorical variables. Treatment effects were analysed using a two-way repeated-measures ANOVA with group (WBPE versus control) as the between-subjects factor and time (baseline versus 5-month follow-up) as the within-subjects factor. Values of partial eta squared (η^2^partial) with 95% confidence intervals were computed to estimate the magnitude of changes in measures over time. Bonferroni-adjusted *post hoc* tests were conducted. Effect sizes were computed as Hedges’ *g* with small-sample correction. For between-group comparisons, *g* was based on pooled s.d. of independent groups. For within-group changes, *g* was calculated using the s.d. of change scores,^16^ with correction for repeated measures. 95% confidence intervals were derived from standard error estimates following Hedges and Olkin.^17^

## Results

The study included two distinct analyses. Analysis 1 (immediate post-treatment effects) evaluated 40 Nova Festival survivors who completed the full WBPE protocol, with symptom severity measured at baseline and immediately after treatment. Analysis 2 (5-month follow-up) involved 45 survivors (27 WBPE completers and 18 wait-list controls), assessed at baseline and at the 5-month follow-up to compare intervention-associated changes with the natural course of symptoms.

### Analysis 1

The WBPE completer sample (*N* = 40) had a mean age of 28.9 years (s.d. = 8.3; range 18–55). Most were male (*n* = 27, 67.5%), born in Israel (96%), unmarried (72.5%) and had at least a high-school education (90%). During the traumatic event, 20% sustained physical injuries, 57.5% lost close friends and 70% reported substance use. During the treatment period, 37.5% reported substance use. None had a prior PTSD diagnosis.

### Primary outcome

#### PTSD symptoms (PDS-5)

As shown in Table 1, WBPE significantly reduced PTSD symptom severity from baseline to post-treatment, with a mean reduction of 15.38 points (s.d. = 14.58), corresponding to a 35.6% improvement (*p* < 0.0001; Cohen’s *d* = 0.97, 95% CI (0.50, 1.44)). The prevalence of probable PTSD (PDS-5 score ≥ 28 (11)) decreased significantly from 77.5% (31/40) at baseline to 42.5% (17/40) at post-treatment (X^2^ = 10.2, d.f. = 1, *p* < 0.0015).

**Table 1:** Changes in PTSD (PDS-5), depression (PHQ-9) and anxiety (GAD-7) symptoms from baseline to post treatment following war-adapted brief prolonged exposure (WBPE) Table 1 long description.

Questionnaire	Baseline *N* = 40	Post WBPE *N* = 40	Statistics
PDS-5 Total score (mean ± s.d)	43.2 ± 16.0	27.8 ± 15.7	*F*(1,78) = 18.75, *p* < 0.0001
Percentage above cut-off (≥ 28)	77.5 (31/40)	42.5 (17/40)	*X* ^2^ = 10.2, d.f. = 1, *p* < 0.0015
PHQ-9 Total score (mean ± s.d)	13.7 ± 5.2	9.8 ± 5.8	*F*(1,74) = 9.0, *p* < 0.004
Percentage above cut-off (>10)	51.28 (20/39)	29.73 (11/37)	Not significant X^2^ = 3.65, d.f. = 1, *p* = 0.056
GAD-7 Total score (mean ± s.d)	13.2 ± 5.6	8.7 ± 4.7	*F*(1,74) = 13.7, *p* < 0.00045
Percentage above cut-off (>10)	69.23 (27/39)	37.84 (14/37)	X^2^ = 7.53, d.f. = 1, *p* < 0.0065

PDS-5, Post-Traumatic Diagnostic Scale; PHQ-9, Patient Health Questionnaire-9; GAD-7, generalised anxiety disorder-7.

*N*s vary because of missing items; denominators are shown.

#### Depression (PHQ-9)

WBPE significantly reduced depressive symptoms from baseline to post-treatment, with a mean reduction of 3.8 points (s.d. = 6.06), corresponding to a 27.8% improvement (*p* < 0.004; Cohen’s *d* = 0.69, 95% CI (0.23, 1.15)). The prevalence of clinically significant depressive symptoms (PHQ-9 score ≥ 10^18^) decreased from 51.3% (20/39) at baseline to 29.7% (11/37) at post-treatment, but this change did not reach statistical significance (χ^2^ = 3.65, *p* = 0.056) (Table 1).

#### Anxiety (GAD-7)

WBPE significantly reduced anxiety symptoms from baseline to post-treatment, with a mean reduction of 4.42 points (s.d. = 6.16), corresponding to a 33.54% improvement (*p* < 0.00045; Cohen’s *d* = 0.85, 95% CI (0.36, 1.30)). The prevalence of clinically significant anxiety symptoms (GAD-7 score ≥ 10) decreased significantly from 69.2% (27/39) at baseline to 37.8% (14/37) at post-treatment (*X* ^2^ = 7.53, d.f. = 1, *p* < 0.0065; Table 1). Overall, immediate post-treatment findings suggest that WBPE was associated with clinically meaningful reductions in PTSD, depression and anxiety symptoms among survivors with acute stress reactions.

### Analysis 2

#### Participant characteristics

In the WBPE group, the mean age was 30.0 ± 9.8 years (± s.d.) (range: 22–55), most participants were male (*n* = 18, 66.7%) and born in Israel (96%). The majority were unmarried (70.4%) and had at least a high-school education (76%). During the traumatic event, 18.5% sustained physical injuries, 44.4% lost close friends or non-first-degree relatives and 70.37% reported substance use. During treatment, 33.33% continued substance use.

The control group had a comparable demographic profile, with a mean age of 29.4 ± 6.8 years (s.d.), predominantly male participants (*n* = 10, 55.6%) and most born in Israel (94.4%). No significant between-group differences were observed in demographic characteristics (Table 2).

**Table 2:** Baseline characteristics of the wait-list control and war-adapted brief prolonged exposure (WBPE) groups Table 2 long description.

Characteristic	Control *N* = 18	WBPE *N* = 27	Statistics
Age (years, mean ± s.d.)	29.4 ± 6.8	30.26 ± 9.8	Not significant
Range	22–46	22–55	
Male (%)	55.6	66.7	Not significant
Born in Israel (%)	94.4	96.2	Not significant
Family status Unmarried (%)	72.2	70.3	Not significant
Substance use during the event (%)	70.59	70.3	Not significant
Substance use after the event (%)	77.78	33.33	χ^2^ = 8.54, d.f. = 1, *p* < 0.004
Visits to therapeutic centres			
Never (%)	11.11	30.77	Not significant χ^2^ = 5.3, d.f. = 2, *p* = 0.071
A few times (%)	44.44	53.85	
Many times (%)	44.44	15.38	
PDS-5 Total score (mean ± s.d.)	48.27 ± 14.26	41.25 ± 15.45	Not significant
Percentage above cut-off (≥28)	94.44	77.78	Not significant
PHQ-9 Total score (mean ± s.d.)	16.44 ± 5.8	13.44 ± 4.29	Not significant
Percentage above cut-off (>10)	83.33	74.07	Not significant
GAD-7 Total score (mean ± s.d.)	15.66 ± 5.79	13.03 ± 5.48	Not significant
Percentage above cut-off (>10)	77.78	66.67	Not significant

PDS-5, Post-Traumatic Diagnostic Scale-5; PHQ-9, Patient Health Questionnaire-9; GAD-7, generalised anxiety disorder-7.

#### Substance use

During the study period, 51.11% of participants (23/45) reported substance use, predominantly cannabis, alcohol, MDMA (known as ecstasy), or combinations thereof. Substance use during the study period was more common in the control group than in the WBPE group (14/18 *v*. 9/27 participants, respectively; Pearson χ^2^ = 8.54, d.f. = 1, *p* < 0.004; Table 2).

#### Therapeutic centres

Visits to these centres were more frequent in the control group than in the WBPE group, although this difference did not reach statistical significance (Pearson χ^2^ = 5.3, d.f. = 2, *p* = 0.071; Table 2).

#### Baseline clinical status

At baseline, there were no significant between-group differences in any clinical questionnaire scores or in the proportion of participants exceeding established clinical thresholds. A high proportion of participants exceeded clinical thresholds for depressive and anxiety symptoms. Clinically significant depressive symptoms were present in 74.07% (20/27) of participants in the WBPE group and 83.33% (15/18) of participants in the control group. Clinically significant anxiety symptoms were present in 66.67% (18/27) of participants in the WBPE group and 77.78% (14/18) of participants in the control group.

### Primary outcome

#### PTSD symptoms (PDS-5)

**Fig. 2:**
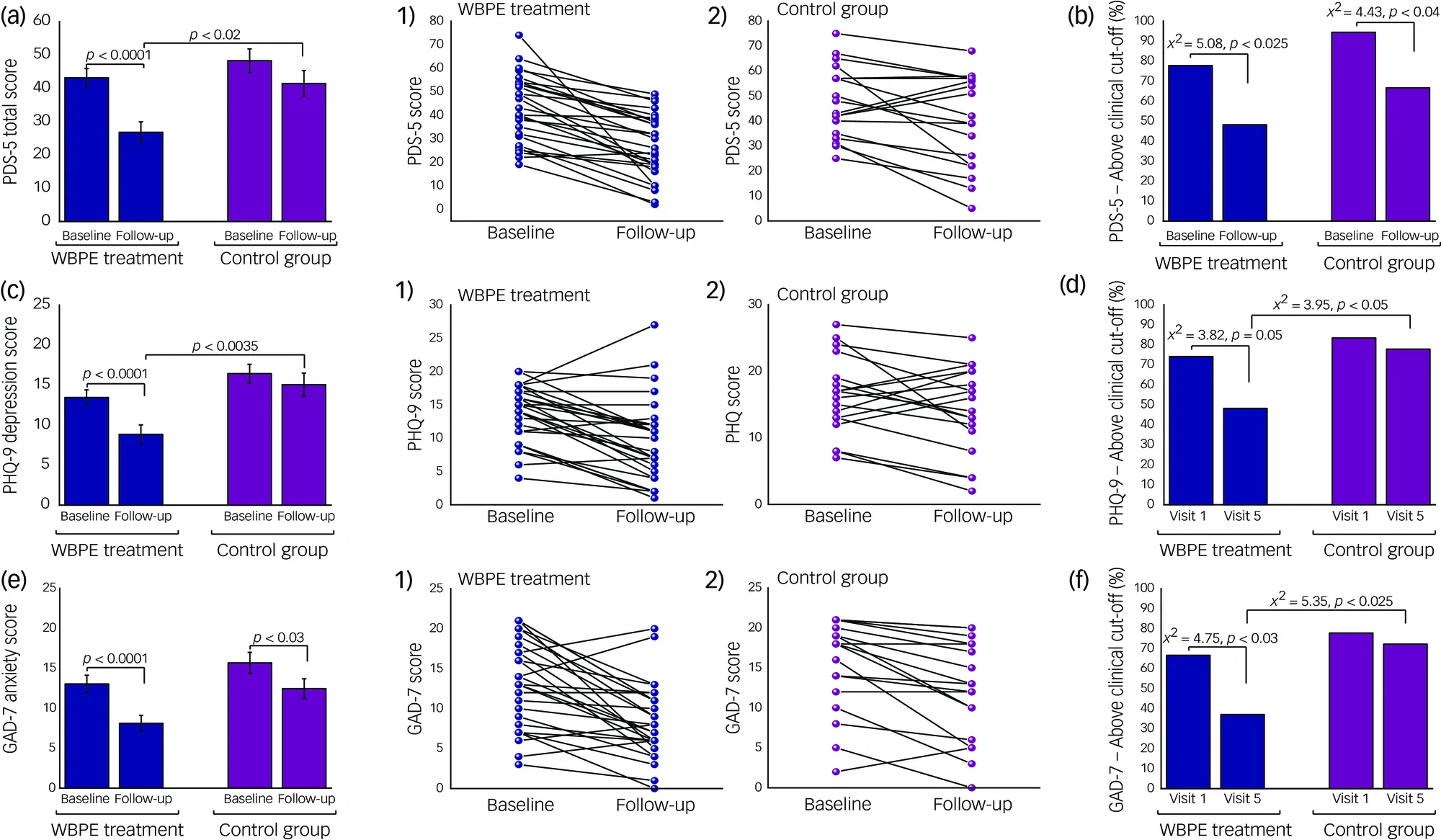
Effects of war-adapted brief prolonged exposure (WBPE) on post-traumatic stress disorder (PTSD (PDS-5)), depression (PHQ-9), and anxiety (GAD-7) at baseline and 5-month follow-up. (a) Mean PTSD symptom severity (PDS-5) at baseline (visit 1) and 5-month follow-up (visit 5) in WBPE participants versus controls. (a1–a2) Individual PDS-5 scores at baseline and follow-up for WBPE and control groups. (b) Proportion of participants scoring above the clinical cut-off on PDS-5 at baseline and follow-up. (c) Mean depression severity (PHQ-9) at baseline and follow-up. (c1–c2) Individual PHQ-9 scores for WBPE and control groups. (d) Proportion of participants above the PHQ-9 clinical cutoff at baseline and follow-up. (e) Mean anxiety severity (GAD-7) at baseline and follow-up. (e1–e2) Individual GAD-7 scores for WBPE and control groups. (f) Proportion of participants above the GAD-7 clinical cut-off at baseline and follow-up. Data are presented as mean ± SEM for continuous scores and percentage prevalence for categorical outcomes. PDS-5, Post-Traumatic Diagnostic Scale-5; PHQ-9, Patient Health Questionnaire-9; GAD-7, generalised anxiety disorder-7.

WBPE significantly attenuated PTSD symptoms, as shown by the change in PDS-5 total severity score from baseline to 5-month follow-up (Fig. 2(a), (a1), (a2)). A repeated measures ANOVA revealed significant main effects of group (*F*(1,43) = 6.4, *p* < 0.02, η^2^partial = 0.13, 95% CI (0.004, 0.315)) and time (*F*(1,43) = 25.4, *p* < 0.0001, η^2^partial = 0.37, 95% CI (0.147, 0.538)), with no interaction effect. *Post hoc* Bonferroni tests confirmed that the WBPE-treated group exhibited a significant reduction in PDS-5 scores from baseline to 5-month follow-up (*p* < 0.0001, Hedges’ *g* = −0.963, 95% CI = (−1.42, −0.51)) and significantly lower scores compared with the control group at 5-month follow-up (*p* < 0.02, Hedges’ *g* = −0.84, 95% CI = (−1.45, −0.23)). The mean change in PDS-5 scores from baseline to 5-month follow-up was −14.48 (s.d. 13.6) in the WBPE group compared with −6.83 (s.d. 14.3) in the control group. These findings indicate that WBPE was associated with a significantly greater reduction in PTSD symptom severity compared with the control condition.

At the 5-month follow-up, probable PTSD, defined as a PDS-5 score ≥ 28,^11^ was observed in 48.15% of participants in the WBPE group (13/27) and 66.67% of participants in the control group (12/18; Fig. 2(b)), a difference that did not reach statistical significance. Although both groups showed reductions in probable PTSD diagnosis rates, the absolute between-group difference was approximately 18.5 percentage points, but the confidence interval for the directional difference crossed zero. A *post hoc* power analysis indicated that the study had only 24% power to detect an effect of this magnitude, and the 95% confidence interval for the between-group difference was wide (–42.9 to 10.5%). Thus, the non-significant categorical finding should be interpreted cautiously, as it may reflect limited statistical power rather than the absence of a clinically meaningful effect.

#### Depression (PHQ-9)

WBPE significantly attenuated depression symptoms, as shown by the change in PHQ-9 total severity score from baseline to 5-month follow-up (Fig. 2(c), (c1), (c2)). Significant main effects of group (*F*(1,43) = 8.9, *p* < 0.005, η^2^partial = 0.17, 95% CI (0.018, 0.360)), time (*F*(1,43) = 17.3, *p* < 0.0002, η^2^partial = 0.287, 95% CI (0.081, 0.468)) and interaction (*F*(1,43) = 4.9, *p* < 0.035, η^2^partial = 0.10, 95% CI (0.000, 0.283)) were observed. *Post hoc* Bonferroni tests showed that the WBPE-treated group exhibited a significant reduction in PHQ-9 scores from baseline to 5-month follow-up (*p* < 0.0001, Hedges’ *g* = −0.95, 95% CI (−1.40, −0.50)) and significantly lower scores compared with the controls at 5-month follow-up (*p* < 0.0035, Hedges’ *g* = −0.96, 95% CI (−1.58, −0.34)). These findings indicate that WBPE was associated with a significantly greater reduction in depressive symptoms compared with the control condition.

At the 5-month follow-up, the prevalence of depression (defined as a PHQ-9 score ≥ 10^18^) in the WBPE group was 48.15% (13/27), which was significantly lower than in the control group (77.78%, 14/18, χ^2^ = 3.95, *p* < 0.05) (Fig. 2(d)). Seven participants (25.92%) in the WBPE group no longer exceeded the PHQ-9 clinical cut-off at follow-up, compared with one participant (5.56%) in the control group.

#### Anxiety (GAD-7)

Significant main effects of group (*F*(1,43) = 5.45, *p* < 0.025, η^2^partial = 0.11, 95% CI (0.001, 0.294)) and time (*F*(1,43) = 33.9, *p* < 0.0001, η^2^partial = 0.44, 95% CI (0.221, 0.593)) were observed (Fig. 2(e), (e1), (e2)), with no interaction effect. *Post hoc* Bonferroni tests revealed significant reductions in GAD-7 scores at the 5-month follow-up compared with baseline for both groups (WBPE-treated: *p* < 0.0001, Hedges’ *g* = −0.76, 95% CI (−1.19, −0.34); Controls: *p* < 0.03, Hedges’ *g* = −0.52, 95% CI (−1.01, 0.04)). Both groups exhibited improvements in anxiety symptoms over time, consistent with the possibility of spontaneous recovery.

At the 5-month follow-up, clinically significant anxiety symptoms were present in 37.04% of the WBPE group (10/27) and 72.22% of the control group (13/18; χ^2^ = 5.35, *p* = 0.021; Fig. 2(f)). Nine participants (33.33%) in the WBPE group no longer exceeded the GAD-7 clinical cut-off at follow-up, compared with two participants (11.11%) in the control group.

### Impact of substance use

**Fig. 3:**
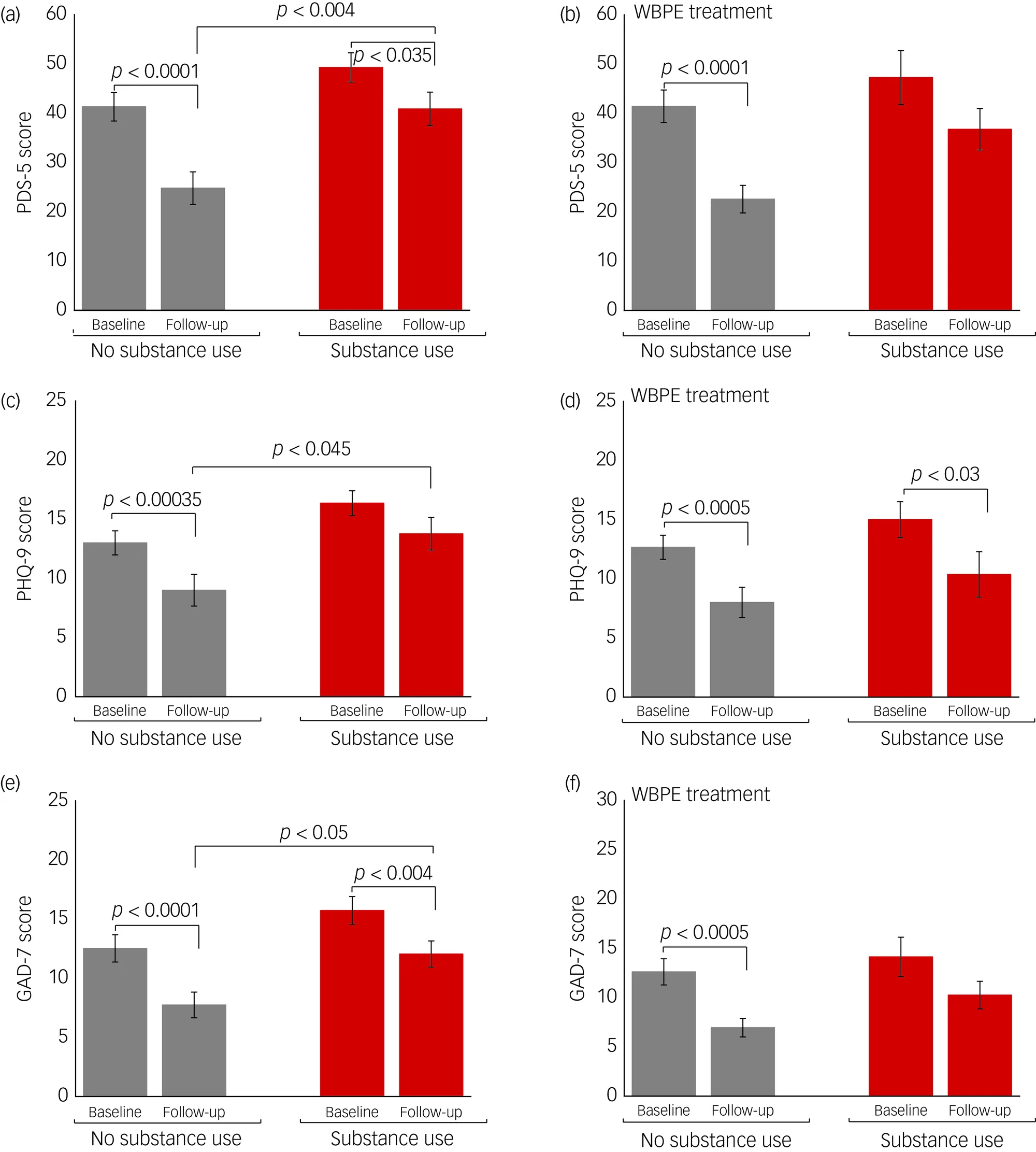
Fig. 3 long description. Impact of substance use on treatment outcomes among Nova Festival survivors. (a) Post-traumatic stress disorder (PTSD) symptom severity (PDS-5) at baseline and follow-up in all participants by substance use status. (b) PTSD symptom severity in war-adapted brief prolonged exposure (WBPE) participants by substance use status. (c) Depression severity (PHQ-9) in all participants by substance use status. (d) Depression severity in WBPE participants by substance use status. (e) Anxiety severity (GAD-7) in all participants by substance use status. (f) Anxiety severity in WBPE participants by substance use status. Data are presented as mean ± SEM. PDS-5, Post-Traumatic Diagnostic Scale-5; PHQ-9, Patient Health Questionnaire-9; GAD-7, generalised anxiety disorder-7.

In the full sample (*N* = 45), participants who reported substance use during treatment or follow-up (‘substance users’) exhibited significantly smaller symptom reductions across all outcome measures at the 5-month follow-up relative to those who reported no substance use (‘non-users’). At the 5-month follow-up, substance users had higher PDS-5 (*p* < 0.004; Fig. 3(a)), PHQ-9 (*p* < 0.045; Fig. 3(c)) and GAD-7 scores (*p* < 0.05; Fig. 3(e)) than non-users. Significant symptom reductions from baseline to 5-month follow-up were observed for PDS-5 and GAD-7 (non-users: both *p* < 0.0001; users: PDS-5 *p* < 0.035, GAD-7 *p* < 0.004). For the PHQ-9, a significant reduction was observed only in non-users (*p* < 0.00035) (Two-way RM-ANOVA: PDS-5: Group: *F*(1,43) = 10.6, *p* < 0.0025, η^2^partial = 0.198, 95% CI (0.029, 0.386); Time: *F*(1,43) = 29.15, *p* < 0.0001, η^2^partial = 0.4, 95% CI (0.177, 0.565); Interaction: *F*(1,43) = 4.1, *p* < 0.05, η^2^partial = 0.087, 95% CI (0.000, 0.264). PHQ-9: Group: *F*(1,43) = 7.0, *p* < 0.015, η^2^partial = 0.14, 95% CI (0.007, 0.328); Time: *F*(1,43) = 20.0, *p* < 0.0001, η^2^partial = 0.32, 95% CI (0.101, 0.493); Interaction: Not significant. GAD-7: Group: *F*(1,43) = 6.8, *p* < 0.015, η^2^partial = 0.14, 95% CI (0.006, 0.325); Time: *F*(1,43) = 37.5, *p* < 0.0001, η^2^partial = 0.47, 95% CI (0.252, 0.612); Interaction: Not significant).

In the WBPE subgroup, significant reductions in PDS-5 (Fig. 3(b)) and GAD-7 (Fig. 3(f)) occurred only among non-users (*p* < 0.00015 and *p* < 0.0005, respectively). PHQ-9 scores improved in both groups, although the reduction was larger among non-users (non-users: *p* < 0.0005; users: *p* < 0.03; Fig. 3(d)) (Two-way RM-ANOVA: PDS-5: Group: *F*(1,24) = 5.3, *p* < 0.035, η^2^partial = 0.18, 95% CI (0.000, 0.421); Time: *F*(1,24) = 22.9, *p* < 0.0001, η^2^partial = 0.49, 95% CI (0.178, 0.662); Interaction: Not significant. PHQ-9: Group: Not significant; Time: *F*(1,24) = 27.8, *p* < 0.0002, η^2^partial = 0.54, 95% CI (0.229, 0.695); Interaction: Not significant. GAD-7: Group: Not significant; Time: *F*(1,24) = 19.6, *p* < 0.0001, η^2^partial = 0.45, 95% CI (0.142, 0.634); Interaction: Not significant). Exploratory analyses indicated that neither sex nor age was significantly associated with changes in clinical outcomes.

Dropout: No significant baseline differences were observed between participants who dropped out and those who remained in the study across demographic or clinical measures. Among participants who discontinued treatment, baseline symptom scores were PDS-5: 45.22 ± 14.3; PHQ-9: 13.33 ± 5.6; and GAD-7: 13.67 ± 5.9.

## Discussion

The present findings provide preliminary evidence that an early-intervention WBPE may attenuate symptoms of PTSD, depression and anxiety, even when implemented under conditions of ongoing wartime threat. Survivors of the Nova Festival massacre who met the criteria for ASD shortly after the trauma received this adapted BPE therapy. Participants who completed WBPE exhibited significantly greater reductions in PTSD and depressive symptom severity both immediately after treatment and at the 5-month follow-up, compared with baseline and with the untreated wait-list control group. These results are consistent with therapeutic benefits that extend beyond spontaneous recovery. Additionally, substance use during the study period, irrespective of treatment assignment, was associated with greater symptom severity across all assessed domains (PTSD, depression and anxiety).

Notably, WBPE yielded these benefits despite ongoing wartime threats, including recurrent missile attacks, daily sirens and persistent trauma reminders such as funerals for friends murdered in the weeks following the Nova Festival massacre. In this context, residual hyperarousal at follow-up should be interpreted cautiously, as vigilance to realistic threats may reflect adaptive threat awareness rather than pathological PTSD symptoms. To our knowledge, this is the first study to examine an early psychological intervention delivered during ongoing trauma. Despite these sustained adversities over more than a year, WBPE was associated with clinically meaningful improvements.

Anxiety symptoms decreased substantially in both groups over time, with no significant between-group differences in continuous GAD-7 scores, suggesting that these improvements may have been largely attributable to natural recovery rather than a specific treatment effect. In contrast, clinically significant depressive symptoms were less frequent in the WBPE group at the 5-month follow-up, with 48.15% exceeding the PHQ-9 clinical threshold compared with 77.78% of controls.

A similar categorical pattern emerged for anxiety, with 37.04% of WBPE participants exceeding the GAD-7 cut-off at follow-up compared with 72.22% of controls. Together, these findings suggest a potential clinical benefit of WBPE, although confirmation in adequately powered randomised controlled trials (RCTs) is required.

The discrepancy between significant between-group differences in continuous PTSD symptom severity and the non-significant categorical difference in probable PTSD rates (48.15% of WBPE participants exceeding the PDS-5 cut-off at follow-up versus 66.67% of controls) may reflect the greater sensitivity of dimensional symptom measures compared with binary diagnostic thresholds, compounded by limited statistical power. Indeed, the *post hoc* power analysis indicated limited power to detect between-group differences in categorical PTSD outcomes. Thus, the absence of a statistically significant difference in probable PTSD rates should be interpreted cautiously and should not be taken as evidence of no clinically meaningful effect. Larger samples are needed to adequately evaluate diagnostic outcomes.

Although early psychological interventions after trauma may alter the trajectory of post-traumatic sequelae and potentially prevent progression from acute distress to chronic impairment, such approaches remain debated.^3^ Numerous studies have failed to support the efficacy of psychological debriefing, the most widely used early intervention, in reducing post-traumatic distress.^19–25^ In most cases, debriefed individuals showed outcomes comparable to those of untreated controls. Methodological limitations in these studies have sustained controversy, particularly given high rates of spontaneous recovery and concerns that premature intervention may disrupt adaptive emotional processing.^3,6,26^

In contrast, controlled trials demonstrate that targeted CBT protocols can reduce PTSD incidence among trauma-exposed individuals, outperforming supportive counselling or wait-list controls. Unlike immediate crisis interventions such as debriefing, trauma-focused CBT is typically initiated weeks to months post trauma and constitutes structured psychotherapy rather than acute emotional support. Systematic reviews and meta-analyses consistently support the potential effectiveness of early trauma-focused CBT, particularly among individuals meeting ASD criteria.^3,27^ Modified prolonged exposure protocols initiated within hours of trauma have demonstrated substantial benefits relative to no-treatment controls in out-patient samples,^28^ though findings have been less consistent in in-patient contexts.^29^

Substance use during the study period merits particular attention. Even moderate use – irrespective of treatment assignment – was associated with greater symptom severity across PTSD, depression and anxiety domains, consistent with the self-medication hypothesis.^30^ One plausible interpretation is that substance use may interfere with recovery-related processes such as emotional processing and fear extinction, which are thought to contribute to natural remission and response to trauma-focused therapies. Post-trauma substance use is often a maladaptive coping strategy to alleviate transient distress, hyperarousal, intrusions, or sleep disturbances. Although bidirectional associations exist between substance use disorders and trauma exposure/PTSD, mediated by shared socio-environmental and neurobiological factors,^31,32^ the observed use here does not necessarily indicate formal disorders. Nonetheless, the observed association with poorer outcomes is consistent with prior evidence suggesting that substance use may disrupt extinction learning, dysregulate stress-related neurobiology, impair fear-modulatory circuits and contribute to poorer sleep, increased avoidance and reduced treatment response.^33–35^ These mechanisms may plausibly contribute to prolonged symptoms and attenuated treatment gains, although they were not directly assessed in the present study.^34^ The higher prevalence of substance use during the study period, but not during the festival itself, in the control group may indicate greater psychological distress among participants who did not receive active treatment. However, this interpretation remains speculative because of the non-randomised design. The findings thus emphasise the importance of integrating systematic substance use assessment and targeted management into post-trauma care, rather than treating it as secondary. Early intervention for substance use, combined with alternative emotion-regulation strategies, could enhance outcomes and promote more durable recovery.

Several methodological limitations should be considered. The absence of random allocation and reliance on self-referral/referral pathways increase the risk of selection bias, and differential follow-up rates between groups may have influenced treatment-effect estimates. The sample was restricted to survivors of a specific civilian mass-trauma event, the Nova Festival attack, limiting generalisability to military personnel, older cohorts, or populations exposed to chronic or recurrent trauma. Substance use was assessed by self-report rather than validated instruments, and the small sample size and frequent polydrug use precluded analyses of substance-specific or dose–response effects. Moreover, the exclusive use of self-report measures, without structured clinician-administered interviews or blinded assessors, constrains diagnostic accuracy. These findings should therefore be regarded as preliminary and require confirmation in adequately powered RCTs.

WBPE was associated with reductions in PTSD, depression and anxiety symptoms that were sustained at 5-month follow-up despite persistent wartime stressors, including recurrent missile threats and pervasive trauma reminders. These findings are encouraging and suggest the feasibility and potential clinical value of early trauma-focused interventions under extreme conditions. Many participants described WBPE as providing valuable, transferable coping skills to manage ongoing stressors.

The association between substance use and poorer outcomes highlights an important clinical consideration, although the mechanisms underlying this association remain to be directly tested. Future research should prioritise large-scale RCTs to rigorously evaluate the efficacy of WBPE, clarify causal effects, refine the protocol for diverse trauma contexts and identify moderators of response, including substance use patterns. Such studies are essential for the development of evidence-based guidelines for early intervention in high-threat environments.

## Supporting information

10.1192/bjo.2026.12116.sm001 Nacasch et al. supplementary material Nacasch et al. supplementary material

## Data Availability

The data-sets used and/or analysed during the current study may be available from the corresponding author on reasonable request.

## Supplementary material

The supplementary material for this article is available online at https://doi.org/10.1192/bjo.2026.12116

## Fig. 3 Long description

Panel A: A bar graph comparing PTSD symptom severity (PDS-5) at baseline and follow-up in all participants by substance use status. The horizontal axis represents substance use status with categories ‘No substance use’ and ‘Substance use’, and the vertical axis represents PDS-5 score ranging from 0 to 60. Bars are vertical and grouped by baseline and follow-up. No substance use shows a decrease from approximately 45 to 25, while substance use shows a decrease from approximately 50 to 40. Panel B: A bar graph comparing PTSD symptom severity (PDS-5) in WBPE participants by substance use status. The horizontal axis represents substance use status with categories ‘No substance use’ and ‘Substance use’, and the vertical axis represents PDS-5 score ranging from 0 to 60. Bars are vertical and grouped by baseline and follow-up. No substance use shows a decrease from approximately 45 to 25, while substance use shows a decrease from approximately 50 to 40. Panel C: A bar graph comparing depression severity (PHQ-9) in all participants by substance use status. The horizontal axis represents substance use status with categories ‘No substance use’ and ‘Substance use’, and the vertical axis represents PHQ-9 score ranging from 0 to 25. Bars are vertical and grouped by baseline and follow-up. No substance use shows a decrease from approximately 15 to 10, while substance use shows a decrease from approximately 18 to 15. Panel D: A bar graph comparing depression severity (PHQ-9) in WBPE participants by substance use status. The horizontal axis represents substance use status with categories ‘No substance use’ and ‘Substance use’, and the vertical axis represents PHQ-9 score ranging from 0 to 25. Bars are vertical and grouped by baseline and follow-up. No substance use shows a decrease from approximately 15 to 10, while substance use shows a decrease from approximately 18 to 15. Panel E: A bar graph comparing anxiety severity (GAD-7) in all participants by substance use status. The horizontal axis represents substance use status with categories ‘No substance use’ and ‘Substance use’, and the vertical axis represents GAD-7 score ranging from 0 to 25. Bars are vertical and grouped by baseline and follow-up. No substance use shows a decrease from approximately 15 to 10, while substance use shows a decrease from approximately 18 to 15. Panel F: A bar graph comparing anxiety severity (GAD-7) in WBPE participants by substance use status. The horizontal axis represents substance use status with categories ‘No substance use’ and ‘Substance use’, and the vertical axis represents GAD-7 score ranging from 0 to 25. Bars are vertical and grouped by baseline and follow-up. No substance use shows a decrease from approximately 15 to 10, while substance use shows a decrease from approximately 18 to 15.

Navigate back to Fig. 3.

## Table 1 Long description

A table comparing PTSD, depression, and anxiety symptoms before and after treatment. The table has 40 participants at baseline and post-treatment. It includes three questionnaires: PDS-5, PHQ-9, and GAD-7. Row 1: PDS-5 Total score, Baseline 43.2 ± 16.0, Post-WBPE 27.8 ± 15.7, Statistics F(1,78) = 18.75, p < 0.0001. Row 2: PDS-5 Percentage above cut-off (≥ 28), Baseline 77.5%, Post-WBPE 42.5%, Statistics X² = 10.2, d.f. = 1, p < 0.0015. Row 3: PHQ-9 Total score, Baseline 13.7 ± 5.2, Post-WBPE 9.8 ± 5.8, Statistics F(1,74) = 9.0, p < 0.004. Row 4: PHQ-9 Percentage above cut-off (>10), Baseline 51.28%, Post-WBPE 29.73%, Statistics Not significant. Row 5: GAD-7 Total score, Baseline 13.2 ± 5.6, Post-WBPE 8.7 ± 4.7, Statistics F(1,74) = 13.7, p < 0.00045. Row 6: GAD-7 Percentage above cut-off (>10), Baseline 69.23%, Post-WBPE 37.84%, Statistics X² = 7.53, d.f. = 1, p < 10.0065.

Navigate back to Table 1.

## Table 2 Long description

The table compares demographic and clinical characteristics between control and WBPE groups. It has 18 rows and 5 columns. The columns are labeled Control, WBPE, and Statistics. The rows include Age, Male, Born in Israel, Family status, Unmarried, Substance use during the event, Substance use after the event, Visits to therapeutic centres, PDS-5 Total score, PDS-5 Percentage above cut-off, PHQ-9 Total score, PHQ-9 Percentage above cut-off, GAD-7 Total score, and GAD-7 Percentage above cut-off. Each row provides specific data for the Control and WBPE groups, along with statistical significance. For example, the mean age for the Control group is 29.4 ± 6.8 years, while for the WBPE group it is 30.26 ± 9.8 years, with the statistics noting it is not significant. The table also includes percentages and scores for various clinical measures, with some showing significant differences between the groups.

Navigate back to Table 2.
