# Supplementary material for: Potential effectiveness of war-adapted brief prolonged exposure for PTSD prevention: preliminary study among Nova Festival survivors during ongoing conflict
Source: BJPsych Open. 2026 Oct 16;12(6):e264. doi: 10.1192/bjo.2026.12116 (PMC13748686; doi:10.1192/bjo.2026.12116)
Supplement: Supplementary file 1 [file S2056472426121164sup001.docx]

**Supplementary Information**

Potential effectiveness of war-adapted brief prolonged exposure for PTSD prevention: A preliminary study among Nova Festival survivors during ongoing conflict

1.1. **Clinical assessments:**

**Posttraumatic Diagnostic Scale** (PDS-5) (Foa et al., 2016): The PDS-5 is a 20-item self-report measure that assesses PTSD symptom severity in the last month according to *DSM-5* criteria. Symptom items are rated on a 5-point scale of frequency and severity ranging from 0 (Not at all) to 4 (6 or more times a week / severe). All questions assess the presence and severity of the PTSD symptoms in relation to the index trauma; symptom questions are based on the DSM–5 symptom clusters intrusion (Items 1–5), avoidance (Items 6 –7), changes in mood and cognition (Items 8 –14), and arousal and hyperreactivity (Items 15–20). The final score is calculated by summing the scores of the individual items, which range from 0 to 80; a higher total score indicates a more severe case of PTSD. A cut-off score of 28 was used to distinguish between persons with a probable PTSD diagnosis (PDS-5 ≥ 28) and persons without a probable PTSD diagnosis (PDS-5 < 28) (Foa et al., 2016).

**Generalized Anxiety Disorder 7 scale (GAD-7)** (Spitzer et al., 2006): The GAD-7 is a well-validated 7-item questionnaire for generalized anxiety disorder, describing the key diagnostic criteria for GAD according to DSM-IV-TR (APA, 2000): Criterion A (fear and anxiety related to a series of events or activities), Criterion B (difficulties in controlling concerns), and Criterion C (anxiety and worry accompanied by at least three additional symptoms, such as restlessness, mild fatigue, difficulty concentrating, irritability, muscle tension, and sleep problems). The GAD-7 assesses the frequency of experiencing the seven core symptoms of GAD over the past two weeks. Response options include 'not at all,' 'on some days,' 'on more than half of the days,' and 'almost every day,' each scored from 0 to 3. The total score ranges from 0 to 21. Using the threshold score of 10, the GAD-7 has a sensitivity of 89% and a specificity of 82% for GAD-7 ([Spitzer et al., 2006](https://www.sciencedirect.com/science/article/pii/S0165032719313643?via%3Dihub#bib0060)). The GAD-7 has demonstrated excellent internal consistency, strong convergent validity, a unidimensional factor structure, and acceptable sensitivity and specificity for detecting generalized anxiety disorder (Pérez-Pedrogo et al., 2022; Plummer et al., 2016; Romano et al., 2022; Spitzer et al., 2006; Sun et al., 2021; Tiirikainen et al., 2019).

**Patient Health Questionnaire 9 (PHQ-9)** (Kroenke et al., 2001): The PH-9 is the 9-item depression module from the full PHQ. As a severity measure, the PHQ-9 score can range from 0 to 27, since each of the 9 items can be scored from 0 (not at all) to 3 (nearly every day). PHQ-9 scores of 5, 10, 15, and 20 represent valid and easy-to-remember thresholds demarcating the lower limits of mild, moderate, moderately severe, and severe depression. In particular, scores less than 10 seldom occur in individuals with major depression while scores of 15 or greater usually signify the presence of major depression (Kroenke et al., 2001).

1.2. **War-Adapted Brief Prolonged Exposure (WBPE)**

The first session begins with an overview of the treatment and the rationale for Brief Prolonged Exposure (BPE). The therapist explains how avoidance maintains PTSD symptoms and impedes recovery, and describes the core therapeutic procedures, namely in vivo and imaginal exposure.

A trauma-specific interview is conducted to identify the index trauma — the most distressing segment of the prolonged traumatic event (which often lasted many hours or even a full day). Because the event extended over a long period, it is essential to pinpoint the specific parts that generated the highest levels of distress and that continue to appear in intrusive symptoms (e.g., flashbacks, nightmares, and intrusive images).

The session also includes psychoeducation about common reactions to trauma and concludes with training in diaphragmatic breathing as a general stress-reduction technique.

The second session introduces both in vivo and imaginal exposure. It begins with a detailed rationale for in vivo exposure. The therapist clarifies that, although the external danger persists due to the ongoing war, many situations and stimuli that serve as trauma reminders are relatively or entirely safe (e.g., listening to trance music, sleeping in the dark, or being in crowded places near shelters). The therapist and patient collaboratively construct a personalized in vivo exposure hierarchy of avoided or distress-evoking situations. Patients are encouraged to engage in gradual exposure to these situations as homework. Given that many survivors experienced significant loss, exposure also addresses reminders of the deceased (e.g., meeting mutual friends or viewing photographs), alongside a gradual return to previously enjoyable activities without excessive guilt.

In the second part of the session, the rationale for imaginal exposure is provided. Imaginal exposure involves revisiting and recounting the traumatic memory aloud, with eyes closed and in the present tense. This is followed by 15–20 minutes of emotional processing, during which negative emotions (fear, anger, guilt, and sadness) are explored and maladaptive cognitions are addressed and modified (e.g., “I am to blame for the death of my friend” or “Freezing means I am weak”).

Imaginal exposure and processing continue in Sessions 3–5. Starting from Session 4, patients are instructed to focus repeatedly on the most distressing parts of the memory (“hot spots”).

Between sessions, patients are assigned daily listening to the imaginal exposure recording and continued practice of in vivo exposures.

**REFERENCES**

Foa, E.B., McLean, C.P., Zang, Y., Zhong, J., Powers, M.B., Kauffman, B.Y., Rauch, S., Porter, K., Knowles, K., 2016. Psychometric properties of the Posttraumatic Diagnostic Scale for DSM-5 (PDS-5). Psychological assessment 28, 1166-1171.

Kroenke, K., Spitzer, R.L., Williams, J.B., 2001. The PHQ-9: validity of a brief depression severity measure. Journal of general internal medicine 16, 606-613.

Lamers, F., Jonkers, C.C., Bosma, H., Penninx, B.W., Knottnerus, J.A., van Eijk, J.T., 2008. Summed score of the Patient Health Questionnaire-9 was a reliable and valid method for depression screening in chronically ill elderly patients. Journal of clinical epidemiology 61, 679-687.

Pérez-Pedrogo, C., Sánchez-Cardona, I., Castro-Díaz, B., López-Torres, S., 2022. Psychometric Properties of the Generalized Anxiety Disorder 7-item Scale in Adolescents: An Effective Screening Tool for School and Community Settings. Puerto Rico health sciences journal 41, 226-232.

Plummer, F., Manea, L., Trepel, D., McMillan, D., 2016. Screening for anxiety disorders with the GAD-7 and GAD-2: a systematic review and diagnostic metaanalysis. General hospital psychiatry 39, 24-31.

Romano, I., Ferro, M.A., Patte, K.A., Leatherdale, S.T., 2022. Measurement Invariance of the GAD-7 and CESD-R-10 Among Adolescents in Canada. Journal of pediatric psychology 47, 585-594.

Spitzer, R.L., Kroenke, K., Williams, J.B., 1999. Validation and utility of a self-report version of PRIME-MD: the PHQ primary care study. Primary Care Evaluation of Mental Disorders. Patient Health Questionnaire. Jama 282, 1737-1744.

Spitzer, R.L., Kroenke, K., Williams, J.B., Löwe, B., 2006. A brief measure for assessing generalized anxiety disorder: the GAD-7. Archives of internal medicine 166, 1092-1097.

Sun, J., Liang, K., Chi, X., Chen, S., 2021. Psychometric Properties of the Generalized Anxiety Disorder Scale-7 Item (GAD-7) in a Large Sample of Chinese Adolescents. Healthcare (Basel, Switzerland) 9.

Tiirikainen, K., Haravuori, H., Ranta, K., Kaltiala-Heino, R., Marttunen, M., 2019. Psychometric properties of the 7-item Generalized Anxiety Disorder Scale (GAD-7) in a large representative sample of Finnish adolescents. Psychiatry research 272, 30-35.
